# Airway management for one lung ventilation during COVID-19 pandemic: a survey within Italian anesthesiologists

**DOI:** 10.1186/s44158-021-00029-0

**Published:** 2022-01-18

**Authors:** Marco Rispoli, Federico Piccioni, Ida Di Giacinto, Gerardo Cortese, Stefano Falcetta, Domenico Massullo, Silvia Fiorelli, Ivana Zdravkovic, Cecilia Coccia, Giulio Rosboch, Antonio Corcione, Massimiliano Sorbello

**Affiliations:** 1 Anesthesia and Intensive Care Unit, Vincenzo Monaldi Medical Hospital, Naples, Italy; 2 Anesthesia and Intensive Care Unit, Department of Critical and Supportive Care, Fondazione IRCCS Istituto Nazionale dei Tumori, Milan, Italy; 3 Anesthesia and Intensive Care, Anestesia e Terapia Intensiva Polivalente, Azienda Ospedaliero Universitaria Sant’Orsola-Malpighi—Alma Mater Studiorum, Bologna, Italy; 4 Anesthesia and Intensive Care, Dipartimento di Anestesia, Rianimazione ed Emergenze AOU Citta della salute e della scienza, Torino, Italy; 5 Anesthesia and Intensive Care, Clinica di Anestesia e Rianimazione Ospedali Riunit, Ancona, Italy; 6 Anesthesia and Intensive Care Medicine, Department of Clinical and Surgical Translational Medicine, Sapienza University of Rome, Sant’Andrea Hospital, Via di Grottarossa 1035, 00189 Rome, Italy; 7 Anesthesia and Intensive Care, Casa di cura Gibiino, Catania, Italy; 8 Anesthesia and Intensive Care, UOC Anestesia, Terapia Intensiva, IRCCS IFO “Regina Elena” National Cancer Institute, Rome, Italy; 9 Anesthesia and Intensive Care, Policlinico San Marco University Hospital, Catania, Italy

**Keywords:** Airway management, COVID-19, Lung separation, Aerosol generating procedures, Bronchial blockers

## Abstract

**Background:**

Airway management for thoracic surgery represents a high risk setting for SARS-CoV-2 infection diffusion due to complex and invasive airway instrumentation and techniques.

**Results:**

An 18-item questionnaire was submitted to the 56 members of the Thoracic subcommittee of the SIAARTI Cardio-Thoraco-Vascular Research Group to provide a snapshot of current situation and national variability of devices and procedures for airway management during the COVID-19 pandemic. The response rate was 64%. Eighty-three percent of anesthetists declared that they modified their airway management strategies. The Hospital Management considered necessary to provide a complete level 3 personal protective equipment for thoracic anesthetists only in 47% of cases. Double-lumen tube and bronchial blocker were preferred by 53% and 22% of responders to achieve one-lung ventilation respectively. Over 90% of responders considered the videolaryngoscope with separate screen and rapid sequence induction/intubation useful to minimize the infection risk. Thirty-nine percent of participants considered mandatory the bronchoscopic check of airway devices. Vivasight-DL was considered comfortable by more than 50% of responders while protective box and plastic drape were judged as uncomfortable by most of anesthetists.

**Conclusions:**

The survey reveals many changes in the clinical practice due to SARS-CoV-2 outbreak. A certain diffusion of new devices such as the VivaSight-DL and barrier enclosure systems emerged too. Finally, we found that most of Italian hospitals did not recognize thoracic anesthesia as a high-risk specialty for risk of virus diffusion.

**Supplementary Information:**

The online version contains supplementary material available at 10.1186/s44158-021-00029-0.

## Introduction

Italy was the second country in the world to be affected by the severe acute respiratory syndrome coronavirus 2 (SARS-CoV-2) pandemic surge. At time of writing, Italy accounted for 236,076 cases of confirmed SARS-CoV-2 multi organ syndrome, coronavirus disease (COVID-19), 32,867 associated deaths with 28,603 health-care providers (HCPs) infected (12.1%) [1].

Airway management in patients affected by COVID-19 is considered a high-risk procedure, most of airway instrumentation maneuvers falling in the list of aerosol generating procedures (AGPs) [2]. Symptomatic COVID-19 patients’ airways may express a viral load up to 60 times more than asymptomatic patients, and proximity of airways during critical procedures exposes the airway management team to highest risk of infection [3]. Such a risk was dramatically increased because of the global shortage of personal protective equipment (PPE) during the pandemic peak, facilitating the spread of the disease within HCPs [4].

During pandemic peak, most of countries suspended elective surgery, while maintaining emergency cases and prioritizing cancer surgery. With the so-called phase-2 re-opening (starting May 4, 2020, in progressive steps in Italy), new challenges had to be faced by healthcare systems, including gradual resumption of elective surgery and maximization of cancer surgery, given the massive waiting lists generated during the lockdown period.

Thoracic surgery represents specific surgical setting at higher infection risk due to more complicated and invasive airway instrumentation, use of bronchoscopy, specific techniques (such as lung separation/isolation), potential for intra- and postoperative air leaks (parenchymal and from drainages), and frequent need for postoperative intensive care treatment [5, 6].

Given the characteristics of this surgical specialty, a survey addressed to anesthetists involved in cardio-thoracic anesthesia as members of the “Società Italiana Anestesia, Analgesia, Rianimazione e Terapia Intensiva” (SIAARTI), Cardio-Thoracic and Vascular Anesthesia Research Group was deemed necessary. The aim was to provide a snapshot of current situation and of the national variability of devices (including PPE) and employed procedures during pandemic in order to address focused recommendations and to design specific procedures to increase both patients’ and HCPS’ safety.

## Methods

An 18-items multiple choice and open answer questionnaire was prepared by the steering committee members of the SIAARTI Cardio-Thoracic and Vascular Anesthesia Research Group and the SIAARTI Airway Management Research Group, basing on previous literature evidence, on national and international guidelines and on national data during pandemic.

Between April 15 and May 15, 2020, the questionnaire was emailed to the 56 members of the SIAARTI Cardio-Thoracic and Vascular anesthesia Research Group considered as representative for national reference centers for thoracic surgery. The survey explored a series of domains: the first one included information concerning the hospitals capacity and location data. Surveyed anesthetists were also asked if the COVID-19 outbreak someway influenced or changed their clinical activity and behavior, including approach to airway management in the specific setting of one-lung ventilation (OLV). Whether thoracic surgical activity during the pandemic was modified or re-organized was also explored in the second domain of the survey.

The third domain was referred to the practical application of the recommendations on COVID-19 safe airway management and their adopted application for OLV procedures, including which devices were used.

In the final section, surveyed anesthetists were asked to evaluate three devices: The Viva-sight double lumen tube (DLT) (Ambu A/S, Ballerup, Denmark) (Additional file 1), the *aerosol box* (Additional file 2), and the plastic drapes (Additional file 3) for healthcare providers protection. A final question was addressed concerning the ideal device that any survey responder would have liked the most to have during the COVID-19 crisis. The survey is available in Additional file 7. The survey was administered through an online questionnaire developed with Google Forms (Google, Mountain View, California, USA). An invitation letter for participating to the survey was sent to the members of the Research Group on April 15, 2020. A reminder letter was sent a week later.

The collected data were analyzed using descriptive statistics calculated on the basis of total answers with Microsoft Excel 17.0 (Microsoft Corporation, Redmond, WA, USA) and SPSS 21.0 (IBM, Armonk, NY, USA).

## Results

A total of 36 questionnaires were answered, with a response rate of 64%.

### Domain 1

The national distribution of the participants showed 47%, 17%, and 36% of responders from the North, Center, and South including Islands of the country respectively. As from the survey, the thoracic surgical activity decreased almost uniformly in the whole country, with total suspension in 8% of cases, reduction greater than 50% in 22% less than 50% in 39% of cases. Thirty-one percent of centers reported no decrease in surgical activity.

### Domain 2: Changes of behavior

Eighty-three percent of surveyed anesthetists declared they modified the airway management strategies and techniques during COVID-19 outbreak. Fifty-three percent of responders preferred a double lumen tube (DLT) to achieve OLV, in the assumption that it was considered to offer a better protection from infection. Twenty-two percent preferred a bronchial blocker (BB), while 25% did not express any preference.

According to responses, only in 47% of cases the Hospital Management considered necessary to provide a complete level 3 PPE (Hazmat suit, goggles/facial shield, filtering facepiece particle, FFP3, or FFP2 respirators) for the anesthetists involved in thoracic anesthesia and surgery, despite the greater risk of AGPs due to the peculiar airway management techniques.

A videolaryngoscope with separate screen (VLS-SS) was considered easy to use and useful to optimize intubation success and protect healthcare providers by 92% of the survey responders. Five percent of them, though recognizing its effectiveness, considered it uncomfortable for clinical use, while 3% did not find any usefulness or clinical benefit in the use of this device.

The use of rapid sequence induction and intubation (RSII) to minimize aerosolization and risk of infection, as from Italian recommendations [7], was considered useful by the 97% of the anesthesiologist, though 14% of them reported some difficulties in the practical actuation of RSII. Only 3% of the responders considered RSII useless.

Using the HEPA (high efficiency particulate air) filter at the “Y” end of the DLT, with the metal stylet coming out of the bronchial lumen (Additional file 4), was considered useful to prevent the spread of droplets during tracheal intubation by 61% of the participants. Among these, the technique was rated as very comfortable, quite comfortable, and uncomfortable by 23%, 36%, and 41% of responders respectively. Thirty-nine of anesthesiologists preferred not to use the HEPA filter during tracheal intubation, but applying it only before ventilation was resumed.

The application of the HEPA filter at the proximal end of the DLT lumen, ventilating the nondependent lung (thus disconnected during OLV) (Additional file 5), was reported as useful by 67% of the responders.

Despite being a high ranked AGP, 39% of participants considered mandatory the bronchoscopic check of the airway device used for lung separation/isolation. Among them, 50% of the sample used a disposable bronchoscope, with 25% switching to disposable because of COVID-19 outbreak and 25% using the reusable one.

Whenever awake, tracheal intubation could not be ruled out, despite being discouraged due to higher risk of aerosolization, 47% of responders used the bronchoscope with a single lumen tube (SLT), followed by use of a BB. Fifty-three percent of responders used awake videolaryngoscopy technique, with SLT and BB in 28% of cases with DLT in 25% of cases. Fifty-three percent of the sample did not express any preference.

In case of tracheal tube exchange using an airway exchange catheter (AEC), 92% of the sample reported to adopt specific behaviors and habits compared to the pre-COVID-19 era (use of PPE, suspension of ventilation, no extra oxygen delivery, use of HEPA filters). Tracheal extubation was performed directly in the operating room according to 92% of responses, in Intensive Care Unit (ICU) in 5% and in the Recovery Room (RR) in 3%. After extubation, patients were reported to be moved to surgical ward (61%), RR (22%), and ICU (17%). Eighty-three percent of hospitals reported to replace the anesthetic machine circuit after each surgical case, while in 11% of cases it was cleaned and sanitized; 6% of responses did not report any specific policy.

### Domain 3: Devices assessment

Use of three devices was explored in the third domain of the survey (Additional file 6).

1. VivaSight-DLT was used at least once by 53% of responders, with a global positive appreciation: its use was reported as comfortable in more than 50% of responses for both tracheal intubation and DLT position check.
2. Use of protective box was reported in 58% of responses. The surveyed anesthetists found this device uncomfortable, especially when performing bronchoscopy or during difficult airway management.
3. Use of plastic drape was reported by 67% of responders. As for protective boxes, in the large majority of responses, it was claimed to be highly unsuccessful and uncomfortable in clinical practice.

### Most desired devices

In order to prevent infection during the COVID-19 pandemic and in the following period (phase II), 50% of the surveyed anesthetists expressed the wish to use regularly the VivaSight-DLT, 23% indicated the wish to acquire a VLS-SS, 11% complained the lack of plastic drapes while remnant 8% would use the protective box. A full and regular supply of PPE was indicated only by 5% of the participants and 3% reported no need for anything else.

## Discussion

Airway management with need for airway separation/isolation and indication for OLV represented *a challenge within a challenge* during COVID-19 outbreak. Intubation and airway management have been ranked within the most significant AGPs [2], and techniques for lung isolation/separation may represent advanced airway management procedures exposing HCPs to higher risk [6, 8].

Data from our survey partially reflect these considerations and the implications for specific surgical area, including how the COVID-19 pandemic induced a change in routine clinical practice. The survey indicates that in Italy there was a 61% global reduction for thoracic surgical activity reflecting the patients overflow, the PPE shortage and—probably—a certain difficulty in maintaining the “elective” oncologic surgical activity despite national healthcare system indications.

Lack of patients’ screening, *COVID-free hospitals*, and separation of infected/clear pathways in COVID-hospitals had also a major role [9].

Eighty-three percent of responders declared some change in their routine clinical practice in choice of endotracheal tube (DLT vs SLT + BB); interestingly, DLT was chosen in 53% of cases; in the assumption, it was considered at lower infection risk if compared to SLT placement followed by use of BB or placement of a DLT over an AEC. These opportunities are considered in different thoracic anesthesia guidelines released during the COVID-19 outbreak [6, 8]; nevertheless, no evidence supports one choice against the other. Theoretically, risk of aerosolization, viral spread, and infection is directly related to airway manipulation and instrumentation, which is intuitively increased if using a BB or a tube exchange procedure. Moreover, in the vast majority of cases, BBs require a bronchoscopic check of the correct positioning, given also the well-known tendency of these devices to dislocation [10]. Despite dedicated ports on airway connectors, with differently effective sealing options, disconnections and bronchoscopy could expose the operator to biological risk. Also, BBs require a certain expertise, and it has been found that at least 15 procedures are necessary to develop a satisfactory and safe level of confidence with these devices [11]. Developing such a skill during a COVID-19 pandemic is someway dangerous, so we strongly discourage adoption of this technique for non-skilled users. On the other hand, use of a DLT might be more challenging in some patients (13.6% difficult intubation, 9% difficult mask ventilation, 2% both in a thoracic anesthesia series of 763 patients) [12], with implications on risk of aerosolization and clinical complications for the patient associated with repeated laryngoscopic attempts [13]. Italian data from a previous survey confirm this observation [14] reporting regular use of BBs in only by 5% of cases [15].

During COVID-19 pandemic, in the present survey, we thus registered a four-time increase of BBs use in thoracic anesthesia practice. We might hypothesize that this behavior reflects the perceived advantage of no need to disconnect the patient from the mechanical ventilator for lung exclusion, with consequent reduction of potential aerosolization. This hypothesis might be confirmed also by the observation that 67% of responders used a HEPA filter at the proximal end of the DLT lumen ventilating the nondependent lung (thus disconnected during OLV). There is no evidence-based data for this technique; despite, it is suggested in COVID-19-related airway management [6, 8] and in different case reports [5, 16, 17].

In lack of specific evidence, we believe that the use of HEPA filters on the DLT lumen of the collapsed lung should be encouraged to minimize aerosolization and infection risk. Similarly, a HEPA filter may be added on the DLT yet during the intubation phase aimed to minimize the risk of aerosolization due to coughing or gagging during intubation attempts but adding a HEPA filter might impede swift airway management, unbalancing the DLT during intubation and complicating the stylet removal during DLT advancement. Such a recommendation was well perceived in our sample and adopted by 97% of responders, despite a reported practical difficulty such as avoiding manual ventilation. Thirty-nine percent of responders did not use a HEPA filter during intubation.

Similarly, VLS-SS was suggested in the same guidelines for airway management in COVID-19 patients RSII [7, 18] including specific thoracic anesthesia recommendations [6, 8]. Furthermore, VLS increases the success rate at first attempt for DLT intubation as reported in a recent meta-analysis by Liu et al. [19]. As from our survey, 97% of responders used a VLS.

Similar changes occurred also for other airway maneuvers: in case of tracheal tube change using an AEC, most of anesthetists reported to adopt specific behaviors and habits compared to the pre-COVID-19 era, with special emphasis on generous upper airways suction, insurance of a deep neuromuscular blockade, generous and rigorous pre-oxygenation, performance of the maneuver with VLS and with preparedness of a backup plan in case of failure.

Use of bronchoscope has been one of the most debated issues during the COVID-19 pandemic, given its potential for aerosolization, especially in the awake and spontaneously breathing patient.

As a matter of fact, use of bronchoscope is a mandatory skill and unavoidable procedure in thoracic anesthesia practice [20]. Recommendations from the Italian Intersociety Consensus on Perioperative Anesthesia Care in Thoracic surgery consider fiberoptic assistance strongly recommended [21]. This indication is someway reflected in our survey: 39% of participants considered mandatory the bronchoscopic check of the used OLV device. Out of any doubt, this data suggests a reduction of use of bronchoscope during the outbreak, relying on the assumption that in experts’ hands the use of bronchoscope could be safely reduced [22]. As a side remark, 50% of our responders used a disposable bronchoscope, and 25% switched from reusable to disposable because of the COVID-19 pandemic, reflecting a growing trend and a different approach to cost-effectiveness of disposable devices [23].

Interestingly, in our sample, whenever seemed unavoidable, awake intubation was performed in 47% of cases with a bronchoscope, whereas 53% of responders used an awake technique with a VLS using either SLT and DLT.

In a Chinese case series during COVID-19 outbreak, the VLS was faster and less skill-requiring than bronchoscope in case of spontaneous breathing intubation [24]. We believe that our data reflect on one hand the familiarity with awake bronchoscopic intubation, and on the other the high skills and confidence of surveyed users, given their daily practice in the specialist field of thoracic anesthesia, including experience with awake VLS intubation.

Questions for the last domain, regarding three specific airway techniques/devices, indicated that 50% of a sample of expert anesthetists was familiar and used the VivaSight-DLT, or barrier enclosure systems such as aerosol boxes and drapes. The VivaSight-DL was perceived as more comfortable in respect of conventional techniques for intubation, reduced need for circuit disconnections and bronchoscopy thanks to the built-in camera. Interestingly, the VivaSight-DLT was listed between desirable items by the thoracic anesthetists enrolled in our survey.

Aerosol box, first described by Canelli and colleagues [25], had a fast-worldwide diffusion due to pricing and availability in face of PPE shortage. Many concerns exist, with special reference to ability of these devices to protect against large droplets but not against aerosols (including concerns for post-procedural cleaning) [26], and our data also add further concerns regarding comfort, ergonomics, and maneuverability especially during advanced/difficult airway management and use of bronchoscope. Similar conclusions might be drawn for plastic covers and drapes [27], which share same limitations as the aerosol boxes plus the superior risk of “secondary aerosolization” upon removal. Despite their large diffusion, also in Italian hospitals and with different variations, we believe that the use of barrier enclosure systems should never substitute an adequate PPE setting, and their use should be prudently avoided until when evidence will support their use.

Only 47% of responders subjectively indicating that their hospitals reported adequate PPE supplies, despite the higher-risk setting represented by thoracic surgery compared to other surgical specialties. This need was clearly addressed in our survey, with 5% of reports indicating scarce supply of PPE, and the perceived need of importance of this level of protection, as indicated in the desirable items section. Not a case, our survey underlines that 6 out 10 Italian hospitals do not recognize thoracic anesthesia as a high-risk specialty in terms of exposure to virus diffusion and risk of infection during airway management.

This survey has several limitations: the response rate was 64%, meaning that we might have missed significant responses; moreover, the sample size was very limited, given that the chosen sample consisted of 56 anesthetists, as they were representatives of the major Italian thoracic surgery centers. Aware of the limits of the real national representativeness of such data, this survey allowed us to promptly photograph the pandemic picture during the early days of the outbreak, at least in high volume centers.

In conclusion, during the COVID-19 outbreak in Italy, DLT were mostly used devices to achieve lung separation/isolation and OLV, but with increased use of BBs if compared with pre-pandemic era. Videolaryngoscopes remain confirmed as preferred and preferable devices for intubation.

Bronchoscopy, though perceived as a high-risk procedure in terms of viral spreading potential, was still largely used either for tube position check or for BBs placement, with a certain trend in reduced use thanks to alternative devices and techniques. A certain diffusion of new devices, such as the VivaSight-DLT and barrier enclosure systems, was observed. The first was highly appreciated and largely used, whereas the latter were mostly deemed uncomfortable and limiting airway maneuvers. Adoption of barrier enclosure systems was also indicated as a consequence to a certain shortage or scarcity in PPE availability, whose importance was largely underlined by the survey responders.

In light of these data, we underline the specificity of thoracic anesthesia as a higher-risk setting in terms of aerosolization and of healthcare providers’ exposure to biological risks.

## Supplementary Information

**Additional file 1.** The VivaSight® Double Lumen Tube.

**Additional file 2.** Intubation protective box – By Ascalon Studios - Own work, CC BY-SA 4.0, https://commons.wikimedia.org/w/index.php?curid=89015486.

**Additional file 3.** Barrier enclosure system: plastic drape during airway management.

**Additional file 4.** Higeh Efficiency Particulate Air filter at the “Y” end of the Double Lumen Tube; note the DLT-dedicates stylet coming out of the bronchial lumen swivel connector.

**Additional file 5.** High Efficiency Particulate Air filter at the end of the Double Lumen Tube lumen corresponding to the nondependent lung, disconnected during One Lung Ventilation.

**Additional file 6.** Percentages of appraisal of “new” devices used during thoracic airway management procedures (from left to right: VivaSight, aerosol box, plastic covers).

**Additional file 7.** Appendix 1.

## Data Availability

The data that support the findings of this study are available upon reasonable request.

## Abbreviations

AEC: Airway exchange catheter
AGP: Aerosol generating procedure
BB: Bronchial blocker
COVID-19: Coronavirus disease
DLT: Double lumen tube
FFP: Filtering facepiece particle
HCP: Healthcare provider
HEPA: High efficiency particulate air
ICU: Intensive care unit
OLV: One-lung ventilation
PPE: Personal protective equipment
RSII: Rapid sequence induction and intubation
RR: Recovery room
SARS-CoV2: Severe acute respiratory syndrome coronavirus 2
SIAARTI: Società Italiana Anestesia, Analgesia, Rianimazione e Terapia Intensiva
SLT: Single lumen tube
VLS-SS: Videolaryngoscope with separate screen
