# Supplementary figures and images for: Airway management for one lung ventilation during COVID-19 pandemic: a survey within Italian anesthesiologists
Source: J Anesth Analg Crit Care. 2022 Jan 18;2:3. doi: 10.1186/s44158-021-00029-0 (PMC8765104; doi:10.1186/s44158-021-00029-0)

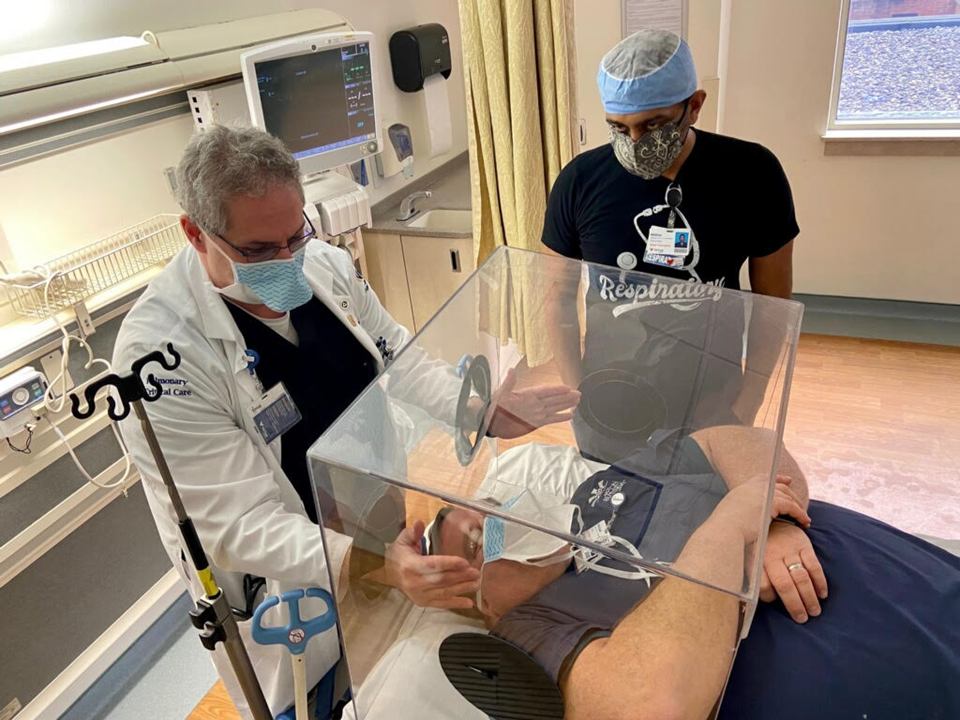

Supplement: Supplementary file 2 — Additional file 2. Intubation protective box – By Ascalon Studios - Own work, CC BY-SA 4.0, https://commons.wikimedia.org/w/index.php?curid=89015486. [file 44158_2021_29_MOESM2_ESM.tif]

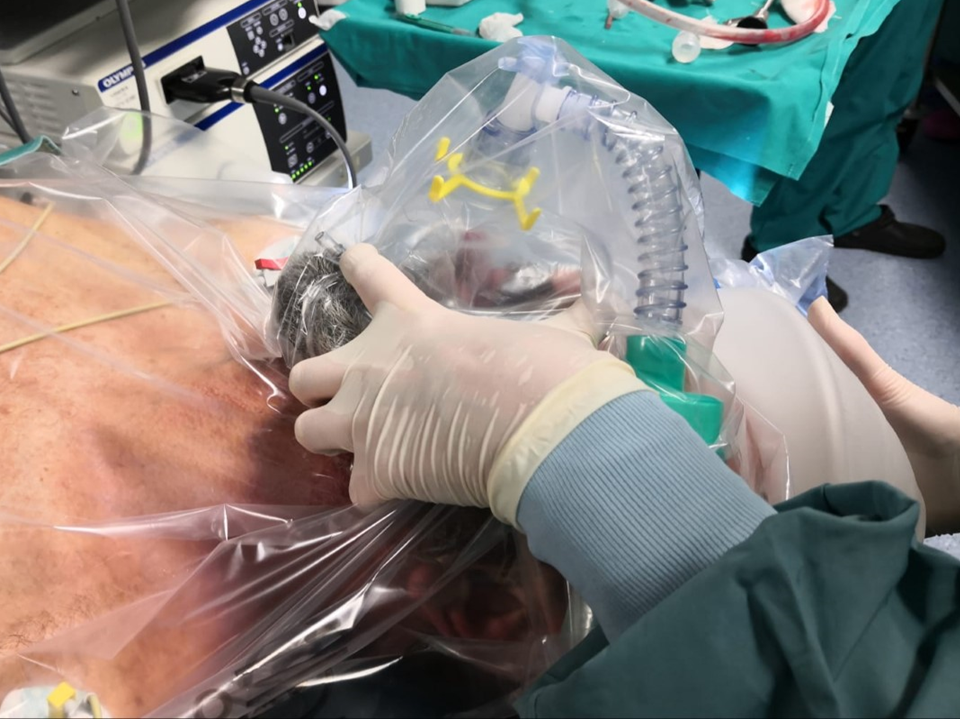

Supplement: Supplementary file 3 — Additional file 3. Barrier enclosure system: plastic drape during airway management. [file 44158_2021_29_MOESM3_ESM.tif]

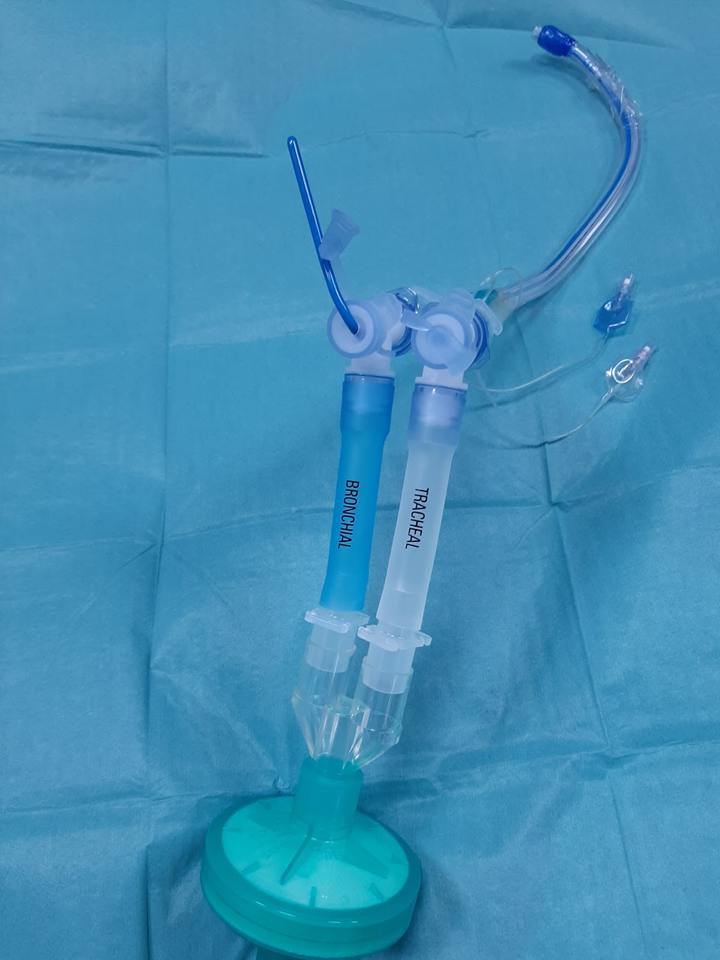

Supplement: Supplementary file 4 — Additional file 4. Higeh Efficiency Particulate Air filter at the “Y” end of the Double Lumen Tube; note the DLT-dedicates stylet coming out of the bronchial lumen swivel connector. [file 44158_2021_29_MOESM4_ESM.tif]

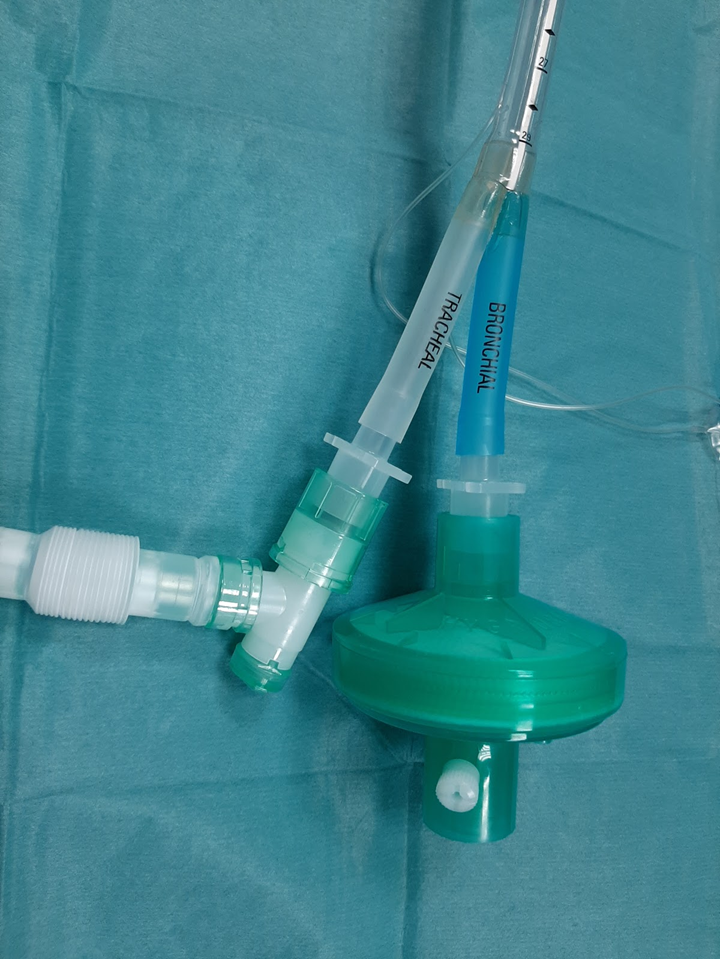

Supplement: Supplementary file 5 — Additional file 5. High Efficiency Particulate Air filter at the end of the Double Lumen Tube lumen corresponding to the nondependent lung, disconnected during One Lung Ventilation. [file 44158_2021_29_MOESM5_ESM.tif]

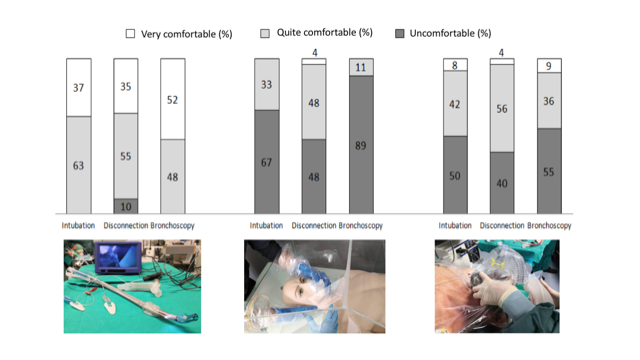

Supplement: Supplementary file 6 — Additional file 6. Percentages of appraisal of “new” devices used during thoracic airway management procedures (from left to right: VivaSight, aerosol box, plastic covers). [file 44158_2021_29_MOESM6_ESM.tiff]
