# Supplementary material for: Airway management for one lung ventilation during COVID-19 pandemic: a survey within Italian anesthesiologists
Source: J Anesth Analg Crit Care. 2022 Jan 18;2:3. doi: 10.1186/s44158-021-00029-0 (PMC8765104; doi:10.1186/s44158-021-00029-0)
Supplement: Supplementary file 7 — Additional file 7. Appendix 1. [file 44158_2021_29_MOESM7_ESM.docx]

**COVID-19 Italian Thoracic Anesthesia Survey**

1. In which hospital do you work?
2. In which city is your hospital located?
3. In your hospital, how much was the elective activity of thoracic surgery reduced during the pandemic?
4. Did you change your routine in the airway management in the operating room during the pandemic?
5. Which One Lung Ventilation device do you consider most protective during the pandemic?
6. Did you use BB before the pandemic?
7. In your hospital, thoracic anesthesia has been assessed at greater risk of air contamination and therefore equipped with adequate PPE (protective suit, FFP3 / 2, face shield) for presumed negative patients?
8. In the current pandemic, how do you consider using the separate screen video laryngoscope (VLS-SS) to reduce the risk of infection?
9. During the current pandemic, how do you consider rapid sequence intubation to reduce the risk of infection?
10. How do you consider the HEPA filter at the tail of the "Y" with a spindle leaving the operating channel of the bronchial lumen?
11. When you use a DLT, do you place a HEPA filter on the non-ventilated lung during exclusion?
12. During the current pandemic, do you use a bronchoscope to check the correct positioning of the DLT or BB?
13. (b) If used, which bronchoscope do you choose?
14. At the end of the surgery, during the current pandemic, where do you normally extubate patients?
15. During the current pandemic, would you adopt specific behaviors and precautions for a possible tracheal tube exchange?
16. During the current pandemic at the end of the surgery, where do you normally transfer patients?
17. At the end of the surgery, how do you manage the ventilation circuit used during the current pandemic?
18. How do you consider for intubation, disconnection and bronchoscopy

- VivaSight DLT
- Protective Box
- Plastic drape

1. Which device you do not currently have in stock would you like to have at your disposal most of all?
